# Supplementary material for: Machine learning to predict microbial community functions: An analysis of dissolved organic carbon from litter decomposition
Source: PLoS One. 2019 Jul 1;14(7):e0215502. doi: 10.1371/journal.pone.0215502 (PMC6602172; doi:10.1371/journal.pone.0215502)
Supplement: S1 Fig — Monte Carlo simulation of the expected number of shared features after sampling from randomly organized sets of 1709 features. (PDF) [file pone.0215502.s005.pdf]

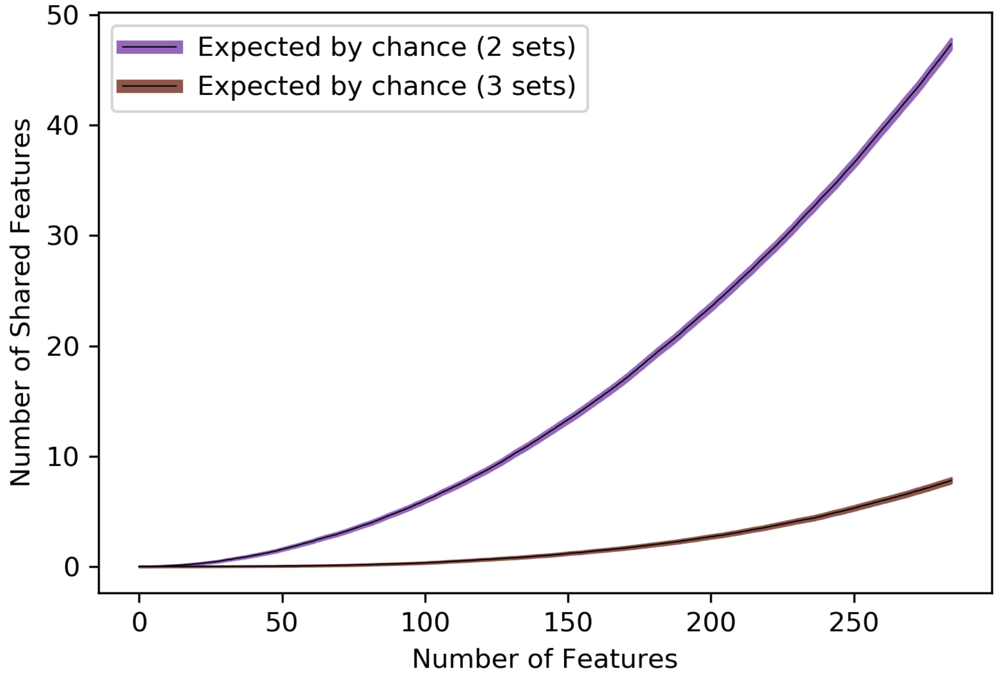

S4 Fig. Monte Carlo simulation of the expected number of shared features after sampling from randomly organized sets of 1709 features. Monte Carlo approach samples features from three randomly organized sets of 1709 features to count the number of features commonly selected in a pair of sets (purple) or within the intersection of all three sets (brown). Plotted curves show the mean and 99% confidence interval from 1,000 simulations as a function of the number of sampled features.
